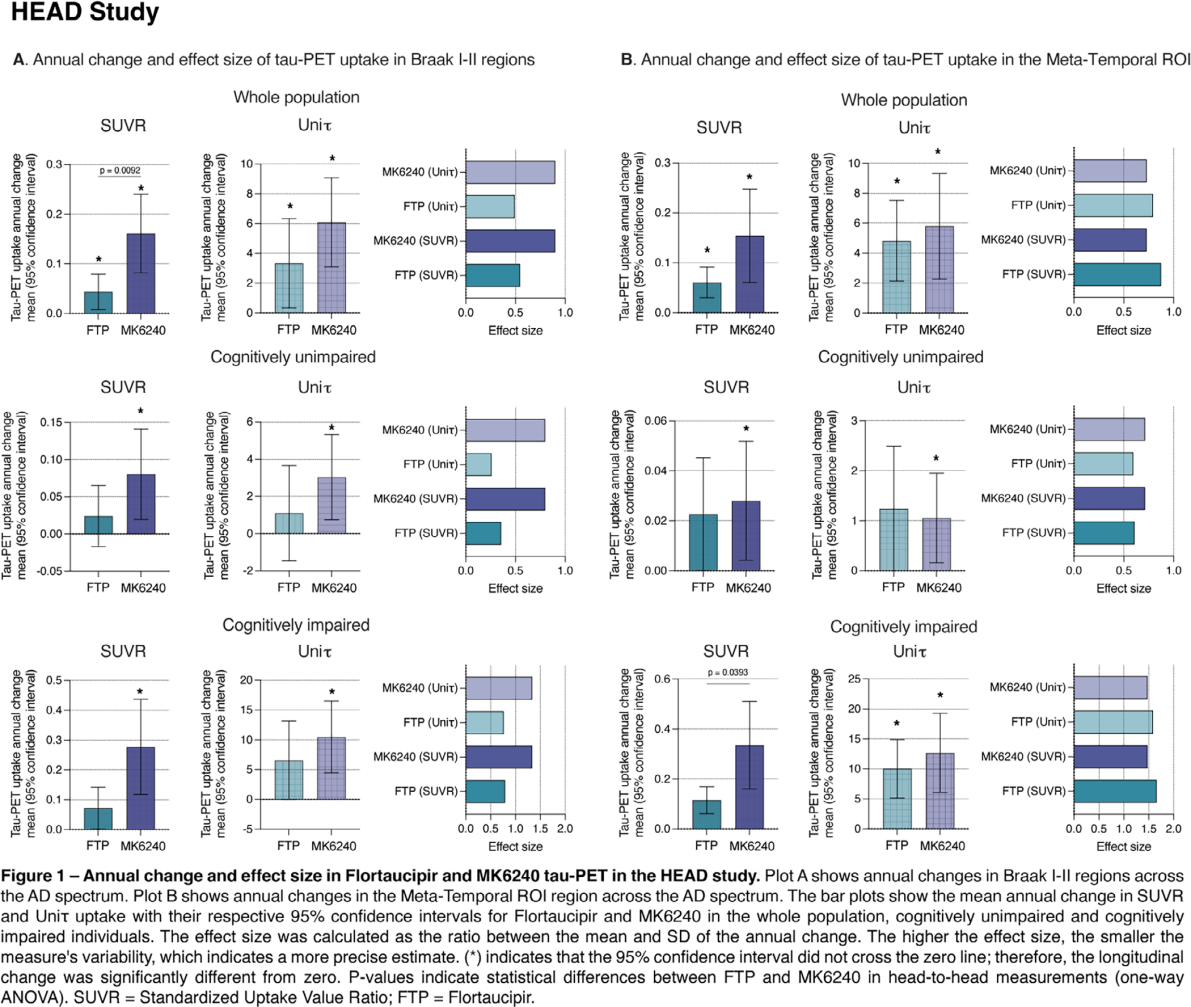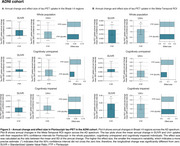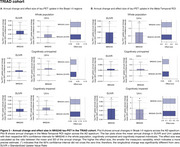# Longitudinal progression and harmonization of tau‐PET tracers

**DOI:** 10.1002/alz70856_105956

**Published:** 2026-01-07

**Authors:** Guilherme Bauer‐Negrini, Pamela C.L. Ferreira, Guilherme Povala, Bruna Bellaver, Firoza Z Lussier, Livia Amaral, Dana L Tudorascu, Quentin Finn, Nesrine Rahmouni, Joseph Therriault, Stijn Servaes, Jenna Stevenson, Arthur C. Macedo, Joseph C. Masdeu, David N. Soleimani‐Meigooni, Juan Fortea, Val J Lowe, Hwamee Oh, Belen Pascual, Brian A. Gordon, Pedro Rosa‐Neto, Suzanne L. Baker, Tharick A Pascoal

**Affiliations:** ^1^ University of Pittsburgh, Pittsburgh, PA, USA; ^2^ Houston Methodist Research Institute, Houston, TX, USA; ^3^ McGill University, Montreal, QC, Canada; ^4^ Memory and Aging Center, Weill Institute for Neurosciences, University of California San Francisco, San Francisco, CA, USA; ^5^ Sant Pau Memory Unit, Hospital de la Santa Creu i Sant Pau, Institut de Recerca Sant Pau ‐ Universitat Autònoma de Barcelona, Barcelona, Spain; ^6^ Mayo Clinic, Rochester, MN, USA; ^7^ Brown University, Providence, RI, USA; ^8^ Washington University in St. Louis, School of Medicine, St. Louis, MO, USA; ^9^ McGill University Research Centre for Studies in Aging, Montreal, QC, Canada; ^10^ Lawrence Berkeley National Laboratory, Berkeley, CA, USA

## Abstract

**Background:**

Tau‐PET tracers have been used to monitor the progression of Alzheimer's disease (AD). However, different tracers present distinct patterns of binding throughout the brain, challenging the harmonization of their findings. Leveraging the HEAD Study, the largest head‐to‐head study of tau‐PET tracers, we recently developed the Uniτ scale, which cross‐sectionally harmonizes Flortaucipir and MK6240 onto a universal tau‐PET measurement. Here, we provide a preliminary evaluation of the Uniτ scale's longitudinal performance in HEAD and two independent cohorts.

**Methods:**

We assessed 422 individuals across the AD spectrum with longitudinal tau‐PET from three cohorts: HEAD [13 cognitively unimpaired (CU), 9 cognitively impaired (CI) individuals, scanned head‐to‐head with Flortaucipir and MK6240], ADNI [74 CU, 208 CI, tracer: Flortaucipir], and TRIAD [72 CU, 46 CI, tracer: MK6240]. Standardized uptake ratios (SUVRs) were harmonized to Uniτ using the Uniτ Ecosystem (unitau.app). Braak I‐II and the Meta‐Temporal regions were used as regions of interest. Annual tau‐PET uptake change was calculated, and the effect size was defined as the mean annual change divided by its standard deviation.

**Results:**

In HEAD, annual change in tau‐PET uptake in Braak I‐II across CU and CI was only detectable in MK6240 (Figure 1A). However, both tracers detected significant annual changes in the Meta‐Temporal ROI for CI (Figure 1B). In both ADNI (Flortaucipir) and TRIAD (MK6240), changes in tau‐PET uptake in Braak I‐II regions were not significant (Figures 2‐3A). However, across all cohorts, the Meta‐Temporal ROI consistently showed detectable annual tau‐PET increases—most pronounced among CI—leading to larger effect sizes than in Braak I‐II (Figures 1‐3B). The Uniτ harmonization did not fundamentally alter the pattern of the findings. However, in ADNI (Flortaucipir), Uniτ yielded a slightly higher effect size than SUVR in CU for both Braak I‐II and Meta‐Temporal regions.

**Conclusion:**

In this large longitudinal sample, our findings confirm that Flortaucipir and MK6240 can detect tau PET changes over time likely associated with the progression of tau tangle pathology. While MK6240 appears to show greater progression in CU, both tracers progress similarly in CI. Our data also suggested that Uniτ harmonized tau PET measurements maintain the longitudinal characteristics of each tau PET tracer for use in clinical trials.